# Childhood and Adult Trauma Experiences of Incarcerated Persons and Their Relationship to Adult Behavioral Health Problems and Treatment

**DOI:** 10.3390/ijerph9051908

**Published:** 2012-05-18

**Authors:** Nancy Wolff, Jing Shi

**Affiliations:** Center for Behavioral Health Services & Criminal Justice Research, Rutgers, The State University of New Jersey, 176 Ryders Lane, New Brunswick, NJ 08901, USA; Email: jshi@cbhs.rutgers.edu

**Keywords:** trauma exposure, childhood trauma, incarcerated men, behavioral health

## Abstract

Rates of childhood and adult trauma are high among incarcerated persons. In addition to criminality, childhood trauma is associated with the risk for emotional disorders (e.g., depression and anxiety) and co-morbid conditions such as alcohol and drug abuse and antisocial behaviors in adulthood. This paper develops rates of childhood and adult trauma and examines the impact of age-of-onset and type-specific trauma on emotional problems and behavior for a sample of incarcerated males (N~4,000). Prevalence estimates for types of trauma were constructed by age at time of trauma, race and types of behavioral health treatment received while incarcerated. HLM models were used to explore the association between childhood and adult trauma and depression, anxiety, substance use, interpersonal problems, and aggression problems (each model estimated separately and controlling for age, gender, race, time incarcerated, and index offense). Rates of physical, sexual, and emotional trauma were higher in childhood than adulthood and ranged from 44.7% (physical trauma in childhood) to 4.5% (sexual trauma in adulthood). Trauma exposure was found to be strongly associated with a wide range of behavioral problems and clinical symptoms. Given the sheer numbers of incarcerated men and the strength of these associations, targeted intervention is critical.

## 1. Introduction

Experiencing physical, sexual, or emotional abuse (referred to as “trauma”) during childhood is known to have predictable immediate and distal impacts on personality development [1,2] Rates of childhood and adult trauma are notably elevated among incarcerated men. In the United States, 1 in 6 state male inmates reported being physically or sexually abused before age 18, and many more witnessed interpersonal violence [3]. Over half of male inmates (56%) reported experiencing childhood physical trauma. By contrast, sexual trauma in childhood is less common (less than 10%) than physical trauma among incarcerated men [4]. Trauma, both experienced and witnessed, often continues into adulthood. Widom and colleagues [5], using a prospective design, found that all types of childhood trauma (physical, sexual, and neglect) elevate the risk of lifetime re-victimization. Repeated trauma over the life cycle also has been found among incarcerated men. For a significant minority of incarcerated men, trauma experiences continued into adulthood but at rates lower than found in childhood, with the exception of being threatened or harmed by a knife or gun, which occurs at roughly equal rates in childhood and adulthood [6]. Trauma experiences, for some incarcerated men, continue inside prison. Six-month prevalence rates of inmate- and staff-on-inmate physical victimization for male inmates were estimated at, respectively, 21% and 25%. Rates of sexual victimization were significantly lower [4].

Emotional abuse, particularly abandonment, is also prevalent among incarcerated men. Over one-quarter of incarcerated men reported being abandoned during childhood or adolescence, diminishing to less than one-fifth (18%) in adulthood [6]. Emotional trauma, considered part of the definition of childhood maltreatment or abuse, is typically defined to occur when the victim is a minor (younger than age 18) and focuses on the failure of a caregiver to meet age-appropriate emotional, physical, or financial needs of a child in his/her care [7]. Childhood emotional trauma may include actions of hostility, rejection, abandonment, or indifference, as well as words that are critical, harsh, belittling, or intended to overpower or intimidate [8]. One form of emotional trauma is abandonment, which occurs when a caregiver deserts a child emotionally, physically, and/or financially [9]. Conceptually, abandonment occurs when the victim is dependent on an adult for his/her survival and, for this reason, is thought to happen only in childhood. For incarcerated people, however, abandonment can occur when the adult is removed from society and placed in a restrictive and resource-constrained environment [6]. The incarcerated person often becomes dependent again on family and friends for financial and emotional resources. Rejection by family members and friends upon incarceration in combination with the social isolation and material deprivation associated with prison may feel like abandonment to incarcerated people and may trigger memories and emotional feelings associated with childhood experiences of abandonment. 

Trauma in childhood, whether physical, sexual, or emotional, has consequences across the life cycle [2,10]. Traumatic abuse that occurred when the victim was a child or adolescent (*i.e*., before age 18) has been found to increase the risk for violent and aggressive behavior and criminality in adulthood [11,12,13]. Childhood abuse also has been found to significantly predict adult arrests for alcohol and/or drug-related offenses [14]. For this reason, it is not surprising to find elevated rates of trauma among incarcerated men. Using a large sample of incarcerated men, Dutton and Hart [15] found that type of abuse experienced in childhood predicted patterns of adult offending, with childhood physical abuse more strongly predicting physical violence and childhood sexual abuse more strongly predicting sexual violence in adulthood. 

The sequelae of childhood trauma, however, expand to behavioral and affective function in adulthood as well. Mental disorders associated with childhood trauma include depression, anxiety disorders, post-traumatic stress disorder, dissociative disorders, and psychosis [16,17,18]. One of the most common mental disorders associated with a history of trauma is depression. Based on a large national survey (the National Co-morbidity Survey), compared to adults with histories of childhood abuse were two and half times more likely to have major depression in comparison to those who had not experienced childhood abuse [19]. Widom, DuMont, and Czaja [20], using data from a prospective longitudinal study, found that children experiencing physical trauma or multiple types of trauma were significantly more likely to have major depression in early adulthood. Similarly, history of childhood sexual abuse has been found to significantly predict mental disorder and mental health problems, controlling for sociodemographic characteristics and other forms of childhood and adult trauma [21]. In a large sample of incarcerated men, higher rates of all types of trauma both in childhood and adulthood were found for male inmates with a self-reported mental disorder compared to their counterparts without a reported mental disorder [6].

Experiencing trauma also contributes to the development of post-traumatic stress disorder (PTSD) [22]. In community samples, approximately 6% of men exposed to trauma develop PTSD; the conditional probability increases to 21% for those experiencing violent trauma (e.g., assaultive violence) [23]. Male inmates have risk factors for developing PTSD including childhood trauma, violent trauma, and multiple trauma exposures [14,15,24]. Compared to their counterparts without PTSD, men with a history of PTSD are more likely to report experiences of drug problems [25]. Regular use of alcohol and illegal drugs is more typical in the lives of abused inmates than those who were not previously abused [3,14]. Exposure to childhood trauma has also been associated with substance abuse in adulthood [26,27]. Regular use of alcohol and illegal drugs is more typical in the lives of abused inmates than those who were not previously abused [3,14]. 

Responses to trauma also include anger, aggression towards others, and self-destructive and suicidal behaviors [28,29,30]. Antisocial personality disorder is also more prevalent among victims of childhood trauma than comparison groups without childhood trauma [31,32]. Johnson and colleagues [33], using a prospective study design, found that childhood neglect predicted personality disorder in early adulthood.

The association between childhood trauma and behavioral health and aggressive behavior in adulthood is well-established in the literature. This research indicates that the timing of abuse (particularly in childhood) and the type of abuse (physical, sexual, emotional) differentially predict psychopathological symptoms in adulthood. These associations have been established in community samples using cross-sectional and longitudinal data. This study extends this research by exploring these associations in a sample of incarcerated male. While it is evident that trauma exposure over the life cycle is prevalent among incarcerated men, the connection between time-dependent (childhood *vs*. adulthood) and specific types of trauma (physical, sexual, and emotional) have not been rigorously investigated. This paper examines general symptoms of psychopathology associated with childhood and adult trauma in an incarcerated male sample. We test the following hypotheses: (1) incarcerated males with trauma exposure will report greater use of behavioral health treatment and psychopathological symptoms/problems than those without trauma exposure; (2) childhood trauma exposure, compared to adult trauma exposure, will more significantly predict psychopathology in adulthood; and (3) types of trauma will have differential impacts on psychopathology in adulthood. 

## 2. Methods

### 2.1. Setting

The study population was drawn from 10 adult prisons for men operated under the auspices of a State Department of Corrections located in the northeast of the United States. The prison system houses approximately 24,000 men in a dozen maximum, medium, and minimum security prisons. The study population included inmates housed at 10 adult prisons for men (excluding the super-max facility and reception center, which are not releasing facilities) that were within 24 months or less of their parole eligibility or maximum sentence date. Excluded from the eligible population were inmates housed in the hospital, administrative segregation, halfway houses, or residential treatment units. Also excluded were individuals off-site on the day of the survey due to court, medical appointments, or work assignments. In addition, individuals were excluded if they were being deported, had detainers for new charges, or otherwise had release eligibility dates that were outside the 24-month window based on recent parole hearings. Roughly 25% of the population was ineligible for these reasons. In all, 7,207 male inmates (three-quarters of the soon-to-be-released population) were eligible to participate. All inmates who met the eligibility criteria (n = 7,207) were invited to participate in a survey about readiness for reentry. The principal investigator provided a 10-minute study orientation to groups of approximately 30 eligible subjects. At the conclusion of the orientation, those interested in participating were consented. Written consent was required to participate in the survey. Subjects were not compensated for participating. Response rates across all facilities ranged from 46.0% to 64.9%, with a mean response rate of 58.4% (SD: 7.3). The consent procedures were approved by Rutgers University Institutional Review Board and the Research Committee of the participating Department of Corrections. 

### 2.2. Instrument

The survey was divided into four parts: reentry readiness and programming, personal well being, abuse history, and background information. The personal well being was measured through ascertain part included questions about current feelings, behavioral health problems and treatment, and problem behaviors in the community prior to incarceration. At the end of the survey instrument, respondents were asked, sequentially, if prior to age 18 (as a minor) or after turning 18 (adult) anyone, including a relative or friend, ever did particular physical or sexual acts to them or made them do these acts to others. 

### 2.3. Procedures

The survey was administered using audio computer-assisted self interviews (A-CASI) and was available in English and Spanish, which accommodated the language needs of the inmate population. Language translation was not a limitation of the study. Subjects responded to a computer-administered questionnaire by using a mouse and followed instructions shown on the screen or provided by audio instructions delivered via headphones. Thirty-three computer stations were available and research assistants were on-site to assist participants as needed. On average, subjects completed the survey within 60 minutes. Data were collected from June 2009 through August 2009.

### 2.4. Participants

A total of 3,986 males (mean age = 33.3, SD = 10.1) aged 18 or older participated in the study. Among the 3,986 male respondents, 2004 (50.3%) were African American, 596 (15.0%) non-Hispanic White, 986 (24.7%) Hispanic, 312 (7.8%) other race or ethnicity (including American Indian (n = 58), Asian (n = 30), Bi-racial (n = 99) and other, unspecified (n = 125), and 88 (2.2%) did not report their race and ethnicity. No statistically significant differences were found between the sample and the eligible population in terms of age, race, or ethnicity. Respondents were excluded from analysis if they did not report victimization experience (n = 91) or other sociodemographic or criminal history variables (n = 109) included in the regression analysis. For all variables used in this analysis, missing data is less than 2.8%. Missing data are treated as missing completely at random (MCAR) and all percentages are based on valid numbers.

### 2.5. Variables

*Sociodemographic.* These variables include age (continuous variable starting at age 18); race/ethnicity (coded categorically with *White, Black, Latino and Other racial group*; Black omitted for comparison) and education (coded categorically with 1 indicating high school graduate/GED or higher and 0 indicating less than high school education). 

*Crime variables.* Two variables were constructed for the offense leading to the current incarceration. They include: sex offense (1 = yes if any of the following: rape, sexual assault or molestation) and violent crime (1 = yes if any of the following: battery, assault, murder, or manslaughter). Time in prison since age 18 was measured in years. 

*Trauma variables.* Six binary trauma variables were constructed: two for each type of trauma: physical, sexual, and abandonment. For each type of trauma, one measured trauma exposure as a minor (younger than age 18) and the other for trauma as an adult (18 and older). Physical trauma was constructed from self-reported responses to questions about whether someone “choked or attempted to drown you; hit you with some object that left welts or caused bleeding; burned you with a match, cigarette, hot liquid, or any other hot object; or threatened or harmed you with a knife or gun.” Sexual trauma was constructed from self-reported experiences where someone “touched, felt, or grabbed you in a way that you felt was sexually threatening; tried or succeeded in getting you to touch their genitals when you didn’t want to; made you have sex by using force or threat of force; or made you have oral or anal sex by using force or threat of force.” Emotional abuse is measured by abandonment. Abandonment was constructed from positive responses to a single question “did anyone ever abandon you?”.

*Depression.* Depression data were collected from two question types; one focusing on treatment and the other focused on symptoms. The treatment question was: “While in prison, have you ever been treated for depression?” and was coded as a binary variable (1 if yes; 0 if no). The four symptom questions asked: “During the past six months, how often have you had: a feeling of …” (hopelessness; helplessness; loneliness; or difficulty caring about anything). Response options were: never, once a month, a few times a month, once a week, a few times a week, every day. The depression symptom variable was the sum of the responses to these questions that were coded as follows: if never, once a month, or a few times a month = 0; if once a week = 1; if a few times a week = 2; if every day = 3 (variable range = 0 to 12).

*Anxiety.* Anxiety data were collected from two question types; one focusing on treatment and the other focused on symptoms. The treatment question was: “While in prison, have you ever been treated for an anxiety disorder?” and was coded as a binary variable (1 if yes; 0 if no). The three symptom questions asked: “During the past six months, how often have you had: a feeling …” (of tenseness or anxiety; that you worry too much; of losing control). Response options were: never, once a month, a few times a month, once a week, a few times a week, every day. The anxiety symptom variable was the sum of the responses to these questions that were coded as follows: if never, once a month, or a few times a month = 0; if once a week = 1; if a few times a week = 2; if every day = 3 (variable range = 0 to 9).

*Substance abuse.* Substance abuse data were collected from questions focusing on treatment and symptoms. The treatment question was: “While in prison, have you ever been treated for a drug or alcohol dependence?” and was coded as a binary variable (1 if yes; 0 if no). The four symptom questions were yes or no responses that asked “In the year prior to coming to prison, did you have a history of …” (using illegal drugs; drinking alcohol to feel better; using prescription drugs that were not prescribed to you; and people complaining about your use of drugs or alcohol) and when summed together ranged from 0 to 4.

*Interpersonal problems.* Data on interpersonal problems were collected from seven questions that asked “In the year prior to coming to prison, did you have a history of …” (getting into fights; having up and down relationships with people; threatening to hurt other people; hitting people when you are angry; not getting along with family members; not having any friends; fighting with strangers). Yes responses were coded as 1 (no responses = 0) and were added together to create a variable ranging from 0 to 7. 

*Self-regulation problems*. Data on self regulation problems were collected from six questions that asked “In the year prior to coming to prison, did you have a history of …” (not being able to keep track of your money; not being able to keep a job; having intensive feelings of anger or rage; doing things impulsively that got you in trouble; gambling; overspending). Yes responses (yes = 1) were summed together, creating a variable ranging from 0 to 6. 

*Aggression.* The Buss-Perry Aggression Questionnaire Short-Form (BPAQ-SF) [34,35] was used to measure aggression. The BPAQ-SF produces a total aggression score. The aggression scale includes 12 questions and the response options are: very unlike me (coded as 1), somewhat unlike me (coded as 2), neither like or unlike me (coded as 3), somewhat like me (coded as 4), very like me (coded as 5). The aggression score is the sum of responses to all questions (total score range = 12–60). Cronbach’s alpha for the BPAQ-SF was 0.89 for this study population.

*Hopelessness.* Hopelessness was measured using The Beck Hopelessness Scale (BHS) [36], a widely used and psychometrically sound measure of hopelessness [37]. The BHS is psychometrically sound with several studies [38,39] supporting the reliability and validity of the measure [40]. For the current study, the Cronbach’s alpha for the BHS was 0.82.

### 2.6. Weighting

Weights were constructed to adjust the sampled population to the full population for different probabilities of selection due to different response rates among facilities and non-response bias. Final weights were rescaled to reflect the actual sample size. Weighted and un-weighted analyses were conducted and because the results were similar, only weighted results are presented. 

### 2.7. Descriptive Analysis

Mean and percentages were estimated based on weighted valid numbers. Respondents were classified into groups according to race and behavioral treatment status. Three trauma groups were identified: physical trauma, sexual trauma, and abandonment and within these groupings, three sub-groups were created for those who reported trauma: as a minor (younger than 18); adult (18 and older), and both as a minor and adult, and one sub-group that reported no trauma. These groups were compared by demographic characteristics and behavioral health treatment while incarcerated. SAS 9.2 was used for all the analysis and Proc survey-means was used to construct all statistics and confidence intervals. The 95% confidence intervals presented in each table are equivalent to two-sided t-test for mean or proportions based on Taylor expansion. The overlap of the confidence intervals between comparison groups suggests that the null hypothesis that the means or proportions are the same between comparison groups at significance level of 0.05 cannot be rejected. 

### 2.8. Statistical Analysis

#### 2.8.1. Missing Data

The loss of information from missing data was small; each variable used in the analysis had a relatively small number of missing data (range: 0.5% to 2.3% with mean = 1.6% and SD = 0.8). Since the missing value proportion is small and consistent (those have missing values for independent variables also had missing values for dependent variables), we used the full sample (n = 3,986) for the regression analysis, resulting in a total loss of 109 observations (2.7%). This approach to handling missing values resulted in all the regression models having the same observations and facilitates cross-model comparisons. 

#### 2.8.2. Hierarchical (Generalized) Linear Model

The sample of inmates in the adult male prisons is nested within facility requiring that we account for the dependence among inmates within the same facility. Not adjusting for the dependence among observations would underestimate the standard error, inflating the significance of the estimators. The hierarchical (generalized) linear (HLM) model adjusts for the clustering within the sample by treating the facility effect as random. Two types of models are estimated. For the binary dependent variables (depression treatment, anxiety treatment, and substance use treatment), the appropriate model is hierarchical generalized linear model with a logit link function. For the continuous dependent variables (depression symptoms, anxiety symptoms, substance use symptoms, interpersonal problems, self-regulation problems, aggression, and hopelessness), HLM without a link function is appropriate. In our preliminary analysis, the level-1 predictors tended to have similar impacts on the outcome variables across facilities. When the level-1 *slopes* are treated as random, the reliability of the estimated parameter is small (<0.05 in most cases), which suggests that the slopes for level-1 predictors have less variability. Hence, the intercept (β_0*j*_) is the only level-1 coefficient treated as random and modeled at level-2. Since there are only 10 male prisons, only one level-2 predictor was possible [41], which was specified as the mean age of the inmate population (W) for each prison. Interaction terms between level-1 and level-2 predictors were not examined for the same reason. All continuous level-1 variables enter the model group-centered (centered on their respective facility mean). Dichotomous predictors are entered in the original format (e.g., white). Centering produces meaningful values for the intercept and facilitates the interpretation of intercept estimates when comparing across facilities. The mathematical formula for the hierarchical generalized linear model is:

Level 1 (continuous dependent variables):


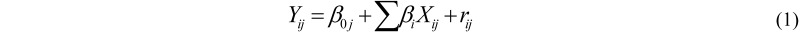


Level 1 (binary dependent variables):


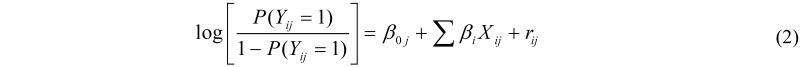


Level 2: 


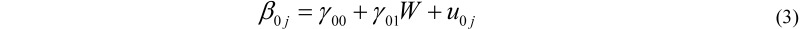


where 


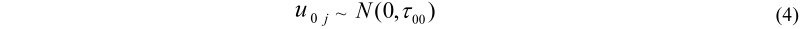


where Y_ij_ represents the scores measured as continuous variables of symptoms or behavioral health problems , while P(Y*_ij_* = 1) represents the probability that inmate i in facility j reported a certain type of treatment, the *β* s represent the level-1 coefficients, in which β_0*j*_ is the intercept of the level-1 equation and X*_ij_* are fixed-effect predictors (e.g., age), β_0*j*_ is the only coefficient modeled in level-2 with intercept *γ*_00_ and predictor W (mean age of inmate population), and *u*_0*j*_ is the level-2 error term, which is assumed to be normally distributed with mean 0 and variance *τ*_00_. The properties of the independent and dependent variables are shown in the appendix.

## 3. Results

### 3.1. Respondents’ Trauma Experience

The trauma experience of male respondents varies by type of trauma and age at time of trauma exposure (see Table 1). Male inmates experienced more physical trauma than sexual trauma independent of age of exposure. Across all types of trauma, trauma exposure rates were higher before age of 18 than in adulthood. White and other racial groups reported experiencing more physical and sexual trauma before the age of 18 than their black counterparts. Hispanic inmates are similar to black inmates in their physical trauma rates, but more similar to White and other racial group inmates in their exposure to sexual trauma.

**Table 1:** Prevalence estimates of trauma experienced by incarcerated men by race, type of trauma and age at time of trauma.

Gender and race/ethnicity	Physical Trauma			Sexual Trauma			Both Physical and Sexual Trauma		
	Minor (age < 18)	Adult (age 18+)	Minor and Adult	Minor (age < 18)	Adult (age 18+)	Minor and Adult	Minor (age < 18)	Adult (age 18+)	Minor and Adult
Male, all (n = 3,895)	44.7 (43.1–46.3)	31.5 (30.0–33.0)	25.1 (23.7–26.5)	10.9 (9.9–11.9)	4.5 (3.9–5.2)	3.7 (3.1–4.4)	9.6 (8.7–10.6)	3.7 (3.1–4.3)	3.2 (2.6–3.8)
White (n = 596)	52.4 (48.4–56.4)	36.4 (32.5–40.3)	29.8 (26.1–33.5)	14.5 (11.6–17.4)	3.9 (2.3–5.4)	2.9 (1.5–4.3)	12.4 (9.7–15.1)	3.0 (1.6–4.4)	2.3 (1.1–3.6)
Black (n = 2,003)	43.6 (41.4–45.8)	31.0 (29.0–33.1)	23.7 (21.8–25.6)	8.5 (7.3–9.7)	3.8 (2.9–4.6)	2.9 (2.1–3.6)	7.5 (6.3–8.7)	2.7 (2.0–3.5)	2.3 (1.6–2.9)
Hispanic (n = 984)	40.0 (36.9–43.1)	28.1 (25.3–30.9)	23.2 (20.5–25.8)	12.1 (10.0–14.2)	5.4 (3.9–6.8)	4.8 (3.5–6.2)	11.0 (9.0–13.0)	5.1 (3.8–6.5)	4.6 (3.3–5.9)
Other ^a ^ (n = 312)	51.9 (46.3–57.5)	35.7 (30.4–41.1)	31.2 (26.1–36.4)	15.9 (11.8–20.0)	8.1 (5.0–11.1)	7.8 (4.8–10.8)	14.0 (10.1–17.9)	6.7 (3.9–9.5)	6.7 (3.9–9.5)

^a^ Other race/ethnicity group includes: American Indian, Asian, Bi-racial, and other, unspecified.

### 3.2. Behavioral Health Treatment and Trauma Experience

As shown in Table 2, overall, men with any type of trauma had higher rates of participation in treatment for depression or anxiety disorder problems (*i.e*., they were overrepresented in treatment) compared to all male inmates without any reported trauma. Among all male inmates, 603/3,895 (15.5%) participated in depression treatment, 291/3,895 (7.5%) participated in anxiety disorder treatment, and 1,026/3,895 (26.3%) participated in drug or alcohol treatment.

**Table 2:** Prevalence estimates of behavioral health treatment by type of trauma and age at time of trauma.

Behavioral health treatment	Physical trauma				Sexual trauma				Abandonment			
	Minor (age < 18) N = 1,741	Adult (age 18+) N = 1,226	Minor and Adult N = 981	No Reported Trauma N = 1,909	Minor (age < 18) N = 415	Adult (age 18+) N = 175	Minor and Adult N = 144	No Reported Trauma N = 3,449	Minor (age < 18) N = 768	Adult (age 18+) N = 712	Minor and Adult N = 445	No Reported Trauma N = 2,860
Depression treatment	21.1 (19.2–23.1)	20.5 (18.3–22.8)	22.2 (19.6–24.8)	11.1 (9.7–12.5)	32.1 (27.6–36.7)	27.4 (20.6–34.2)	27.6 (20.1–35.2)	13.7 (12.5–14.8)	26.9 (23.7–30.0)	25.9 (22.6–29.1)	29.0 (24.7–33.3)	12.3 (11.1–13.5)
Anxiety disorder treatment	10.9 (9.4–12.4)	11.2 (9.4–13.0)	12.5 (10.4–14.6)	4.9 (3.9–5.9)	16.1 (12.5–19.8)	16.4 (10.7–22.0)	15.8 (9.6–21.9)	6.5 (5.7–7.3)	14.1 (11.6–16.6)	14.0 (11.4–16.6)	16.2 (12.7–19.7)	5.6 (4.8–6.5)
Drug or alcohol treatment	28.7 (26.6–30.9)	30.1 (27.5–32.7)	30.2 (27.3–33.1)	23.9 (22.0–25.8)	32.3 (27.8–36.9)	27.6 (20.9–34.3)	28.2 (20.8–35.7)	25.7 (24.2–27.2)	29.6 (26.3–32.8)	34.5 (31.0–38.0)	33.4 (29.0–37.8)	24.6 (23.1–26.2)

### 3.3. Behavioral Health Problems Related to Trauma Experience

Bivariate associations between behavioral health problems and trauma experience may be conflated with other factors affecting behavioral health such as offense type. For this reason, multivariate analyses were conducted and are shown in Table 3 and Table 4. Each behavioral health problem model was estimated using the same set of independent variables to cross-check the evidence. 

**Table 3:** Coefficients and odds ratios for behavioral health problems by individual characteristics of incarcerated men.

Independent variables		Incarcerated Men (N = 3,877)					
		Depression treatment ^a^	Depression symptoms	Anxiety treatment ^a^	Anxiety symptoms	Substance abuse treatment ^a^	Substance abuse problems
Level 2 (Reliability)		0.214	0.843	0.001	0.014	0.789	0.677
Mean age of inmates in each prison		1.03 *	−0.02	1.07 *	−0.02 *	1.02	−0.005
Level 1							
	Age	1.01	−0.01 *	1.00	−0.03 **	1.01	−0.009 *
	Race: White	2.66 **	0.79 **	4.66 **	0.56 **	1.81 *	0.63 **
	Latino	1.30 *	0.46 **	1.23	0.16	0.83 *	0.009
	Other ^b^	1.52 *	0.39 *	1.47	0.25	0.87	−0.06
	Education (>high school/GED *vs*. less)	0.80 *	−0.47 **	0.98	−0.24 *	0.91	−0.11 *
	Time incarcerated since 18	1.00	0.02 *	1.01	0.02 *	1.04 **	0.03 **
	Type of crime: Sex Crime	0.96	−0.15	0.73	−0.22	0.52 *	−0.67 **
	Violent Crime	1.00	−0.27 *	1.25	−0.15	0.74 *	−0.002
	Physical trauma <18 (Yes = 1)	1.40 *	−0.003	1.42 *	0.17	1.03	0.14 *
	Sexual trauma <18 (Yes = 1)	2.06 **	0.44 *	1.52 *	0.38 *	1.41 *	0.31 **
	Physical trauma 18+ (Yes = 1)	1.04	0.40 *	1.22	0.28 *	1.12	0.33 **
	Sexual trauma 18+ (Yes = 1)	0.87	0.39	1.30	0.39	0.81	0.28 *
	Abandoned <18 (Yes = 1)	1.42 *	0.60 **	1.35	0.48 **	0.89	0.11
	Abandoned 18+ (Yes = 1)	1.44 *	0.92 **	1.43 *	0.68 **	1.50 **	0.31 **
Test Statistics							
Chi-square		9.68	45.84	5.32	7.31	62.94	32.81
Df		8	8	8	8	8	8
p-value		0.288	<0.001	>0.5	>0.5	<0.001	*p* < 0.001

^a^ These three models are generalized hierarchical linear model with binary outcomes, odds ratios were reported in those models;

^b^ Other race/ethnicity group includes: American Indian, Asian, Bi-racial, and other, unspecified; * *p* < 0.05, ** *p* < 0.001.

*Behavioral health treatment and symptoms.* Results for the treatment and symptoms models appear in Table 3. At the facility level, the likelihood of receiving depression and anxiety treatment varied significantly among facilities. Facilities with older inmates were more likely to have inmates receiving treatment for depression and anxiety. Demographic variables predicted behavioral health treatment and symptoms. Older incarcerated men were less likely to have depression, anxiety symptoms, or substance abuse history. White incarcerated men, compared to their Black counterparts, were near or more than twice as likely to receive treatment for depression, anxiety disorder, or substance abuse problems while incarcerated. Consistent with their greater use of treatment, they also reported experiencing more behavioral health symptoms, particularly depression, anxiety symptoms, and a history of substance abuse problems in the community. Incarcerated men with GED or higher education consistently had less mental health symptoms and less serious substance abuse history. Criminal history predicted symptoms and treatment as well. Incarcerated men who committed a violent crime were less likely to report receiving substance abuse treatment while incarcerated. Similarly, incarcerated men who committed a sex crime reported fewer problems with substance abuse and were less likely to receive substance abuse treatment while incarcerated. Overall, the more time incarcerated men spent time in prison since age of 18, the more they reported symptoms of depression and anxiety and substance abuse problems in the community. Everything else equal, they are also more likely to have received treatment for substance abuse in prison. 

**Table 4:** Coefficients for behavior problems of individual characteristics of incarcerated men.

Independent variable		Incarcerated Men (N = 3,877)			
		Interpersonal Problems	Self-regulation Problems	Aggression	Hopelessness
Level 2 (Reliability)		0.366	0.030	0.001	0.567
	Mean age of inmate population	−0.03 **	−0.004	−0.25 **	0.004
Level 1					
	Age	−0.04 **	−0.03 **	−0.26 **	−0.02 *
	Race: White	0.51 **	0.34 **	1.47 *	1.09 **
	Latino	−0.03	−0.03	−1.22 *	0.47 **
	Other ^a^	0.15	−0.03	0.04	0.50 *
	Education (>high school/GED *vs*. less)	−0.23 **	−0.22 **	−1.19 *	−0.55 **
	Time incarcerated since 18	0.04 **	0.04 **	0.22 **	0.04 **
	Type of crime: Sex Crime	−0.28 *	−0.73 **	−1.08	−0.09
	Violent Crime	0.58 **	0.03	2.35 **	−0.10
	Physical trauma <18 (Yes = 1)	0.48 **	0.44 **	2.70 **	−0.17
	Sexual trauma <18 (Yes = 1)	0.34 *	0.35 *	0.42	0.55 *
	Physical trauma 18+ (Yes = 1)	0.65 **	0.45 **	2.48 **	0.59 **
	Sexual trauma 18+ (Yes = 1)	0.52 *	0.46 *	1.54	0.85 *
	Abandoned <18 (Yes = 1)	0.27 *	0.28 *	1.79 *	0.44 *
	Abandoned 18+ (Yes =1)	0.51 **	0.42 **	1.55 *	0.42 *
Test Statistics					
Chi-square		12.09	5.01	4.30	21.12
Df		8	8	8	8
p-value		0.147	>0.5	>0.5	0.007

^a^ Other race/ethnicity group includes: American Indian, Asian, Bi-racial, and other, unspecified; * *p* < 0.05, ** *p* < 0.001.

Trauma exposure elevated the likelihood of depression and anxiety treatment and intensified corresponding symptoms. Childhood physical trauma positively predicted depression and anxiety treatment and substance abuse problems, while physical trauma in adulthood independently predicted higher levels of depression and anxiety symptoms, and substance abuse problems. Sexual trauma was most predictive when it occurred in childhood. Childhood sexual trauma positively predicted all types of treatment and behavioral health symptoms, while adult sexual trauma predicted only substance abuse problems. Abandonment in adulthood consistently predicted higher levels of all types of treatment and behavioral health symptoms. Childhood abandonment positively predicted depression treatment and symptoms and anxiety symptoms. 

*Other psychopathological problems.* Table 4 shows the relationships of other behavior problems with trauma variables, controlling for demographics and criminal history. At the facility level, interpersonal and aggression problems varied significantly among facilities, with prison populations with older incarcerated men having less problems. At the individual level and for all models, older incarcerated men, in general, reported less psychopathology. White incarcerated men compared to their Black counterparts reported more psychopathological problems in all areas, while Hispanic incarcerated men, on average, compared to Black reported less aggressive but more hopelessness. Incarcerated men with GED or higher education, compared to those with less education, reported less psychopathology. Convictions related to sex offenses predicted fewer problems with interpersonal relationships and self-regulation. Incarcerated men with violent offenses reported more aggression and interpersonal problems. Time in prison since age of 18 was positively associated with all types of psychopathology. 

Trauma exposure, in general, was positively associated with psychopathology. All types of trauma, experienced in childhood or adulthood, independently and significantly predicted interpersonal and self-regulation problems. Physical trauma (not sexual) and abandonment, in childhood and adulthood, were positively associated with aggressive behavior. All types of trauma experiences (with the exception of physical trauma before age of 18) increased the feeling of hopelessness among incarcerated men.

## 4. Discussion

This study sought to test whether (1) incarcerated males with trauma exposure reported greater use of behavioral health treatment and presence of more psychopathology than those without trauma exposure; (2) childhood trauma exposure significantly predicts psychopathology in adulthood; and (3) types of trauma have differential impacts on psychopathology in adulthood. We found confirmation for each of the hypotheses. 

In general, exposure to trauma elevated the likelihood of receiving behavioral health treatment and the level of psychopathology in adulthood. Our finding of a positive association between trauma exposure and behavioral health symptoms and problems is consistent with previous research based on community [16,18,19,20,21,33,42,43] and incarcerated [15] samples. New to the literature is the finding that trauma exposure significantly increases the likelihood of that men receiving treatment for depression, anxiety, and substance abuse problems while in incarcerated, controlling for demographic and criminal history characteristics. 

In keeping with the literature, the marginal impact of trauma exposure depends on its age of occurrence and type. Childhood trauma, in particular, had strong predictive power. Compared to their counterparts without reported childhood trauma exposure, incarcerated men reporting childhood sexual trauma were twice as likely to report receiving treatment for depression and one and a half times more likely to report receiving treatment for anxiety and substance abuse while incarcerated. They also reported significantly more symptoms of depression, anxiety, substance abuse, interpersonal, and self-regulation problems, and feelings of hopelessness. In contrast with findings reported by Dutton and Hart [15], childhood sexual abuse did not elevate reported symptoms of aggression, after controlling for demographic and criminal history characteristics. 

Childhood physical abuse, on the other hand, inconsistently predicted treatment or clinical symptoms but more strongly predicted other psychopathology, particularly problems with interpersonal, self-regulation, and aggression, which is generally consistent with the evidence in support of the cycle of violence [15,44]. Research on the long term effects of childhood emotional trauma consistently reproduces findings of associations between childhood emotional trauma and psychopathology in adulthood [2]. Using abandonment prior to age 18 as a measure of childhood emotional trauma, we found that childhood abandonment was positively associated with depression treatment, depression and anxiety symptoms, and all four types of psychopathology: interpersonal, self-regulation, aggression, and hopelessness, which replicate findings based on community samples of adults [8,33,42,45,46]. 

The impact of trauma exposure in adulthood on behavioral health problems in adulthood has not been investigated to the extent of as fully as childhood trauma exposure on adult behavioral health, or in terms of its marginal impact on adult problems controlling for childhood trauma of particular types. Our findings indicate that adult trauma exposure has an independent and significant impact on adulthood psychopathology. All forms of adult trauma (physical, sexual, and emotional) were significantly associated with psychopathological problems, and the marginal impact of adult trauma was often equal or greater in magnitude relative to the impact of childhood trauma exposure. Note, for example, that the marginal impact of emotional trauma (abandonment as an adult) on psychopathology in adulthood was frequently larger in magnitude than the impact estimated for childhood sexual or physical trauma. This finding may be unique to incarcerated populations in part because of their greater exposure to violence in their communities prior to incarceration, in part because incarceration is often coincident with emotional and physical separation from family members and friends, and in part because the nature of the crime may cause the family to reject the incarcerated person [6].

Our findings based on an incarcerated sample, confirm previous primarily on community-based research samples, showing that childhood trauma has strong negative associations with adult psychological wellness and that different types of childhood trauma have are independently associated with impacts on adult psychopathology. We extend the knowledge base with findings that indicate a significant impact of (a) all forms of childhood trauma on use of behavioral health treatment during incarceration and (b) adult trauma on adult psychological symptoms and problems for an incarcerated male sample.

While our findings are generally consistent with the extant literature, several limitations warrant mention. First, our data on trauma and psychopathology are based on a cross-sectional design and, as such, our findings indicate associations only, not causal relationships. We cannot confirm or refute that exposure to trauma causes psychopathology. There is, however, consistent evidence that time-dependent and type-specific trauma exposure is associated with a range of depressive and aggressive psychopathologies that are connected etiologically to experiences of trauma. Future research is needed to test for causal sequencing, which in the absence of a prospective design, could be explored by screening incarcerated men for post-traumatic stress disorder, a disorder causally related to trauma exposure. The associations, however, reported herein shed important preliminary light on the nature and extent of trauma within a large sample of incarcerated men, and its relationship to treatment seeking and symptoms of psychopathology. 

Second, sample bias is possible. Our sample excluded certain types of inmates (e.g., inmates housed in the hospital, administrative segregation, halfway houses or residential treatment units). Hence, our research findings apply only to the population of incarcerated men who are held in general population. People who decline to participate in research may be different from those who participate. Identifying these differences is challenging because refusers decline to be interviewed. We did compare the demographic characteristics of refusers to participants. Nonrepresentativeness was tested in terms of gender, age, and ethnicity (no significant differences were found) and adjusted for in the weighting strategy. Yet these characteristics and adjustments may not fully predict variation between the sample and the population. We account for this uncertainty by estimating confidence intervals that provide a reasonable (95%) approximation of the range of variation for the measures. Third, biased reporting may have occurred. Audio-CASI is the most reliable method for collecting information about activities or events that are shaming or stigmatizing. By using audio-CASI, we minimized the motivation to not reveal trauma exposure. To limit mono-method bias among respondents, we used standardized instruments (Buss-Perry Aggression Scale and Beck Hopelessness Scale) that have strong psychometric properties. Our measures of interpersonal and self-regulation problems are not based on scales; rather, they are based on groupings of responses to questions that focus on these problem areas. These groupings of problems are suggestive of problems of getting along with others or regulating particular problematic behaviors. Fourth, emotional abuse is measured by abandonment. The survey instrument included one question on abandonment: “Did anyone ever abandon you?” We did not define what abandonment meant, nor did we use a series of questions that relate to abandonment to form a construct measure. We left it unspecified because, during the pilot testing of the survey instrument, a group of previously incarcerated men living in a halfway house noted that we did not ask about abandonment. Examples of abandonment provided were adoption, foster care, left with strangers or in public places, emotional rejection, failure to communicate, rejection when convicted, as well as others. Given that “abandonment” is identified in the literature as a form of emotional abuse, we added a single question to our survey to test its relevance. The predictive strength of abandonment on psychopathology suggests an area of future research that would deconstruct the meaning of abandonment for this population and construct a scale that captured its meaning. 

Despite these limitations, the present study has important implications for practice and suggests areas for future research. First, on the practice side, our findings suggest the importance of screening for trauma-related disorders (particularly PTSD) and to provide trauma-informed treatment for incarcerated men. There are several effective strategies for trauma-related behavioral health, including Seeking Safety [47,48] and Trauma Recovery and Empowerment Model (TREM) [49]. Both of these interventions have been found generally effective (in terms of reductions in PTSD symptoms) in quasi-experimental or small pilot studies, often without randomization. And on the research front, areas of new investigation would explore the meaning and impact of abandonment on the psychopathology of inmates and the connections among trauma exposure, psychopathology, and criminal behavior in an effort to prevent future criminality. Our findings also warrant replication in other male incarcerated samples (to test for generalizability), as well as among male samples drawn from juvenile detention and probation. Since our findings only pertain to incarcerated men, it is also important to know whether and how associations among trauma and psychopathology may differ for incarcerated females.

## 5. Conclusions

Research evidence consistently finds elevated rates of trauma among incarcerated samples. Our study provides evidence that trauma exposure among incarcerated men is associated with a wide range of behavioral problems and clinical symptoms. Given the sheer numbers of incarcerated men and the strength of these associations, targeted intervention is critical. The first next step is treatment for trauma and its consequences inside correctional settings. This is particularly important for incarcerated men—a largely neglected population. Historically, research attention has focused on the psychological needs and problems of incarcerated women, not those of men. This study and its array of findings is a call to action; action to screen for and treat trauma-related disorders and behaviors among incarcerated men and to study the effectiveness of trauma-informed interventions on psychological and criminal justice (e.g., recidivism) outcomes. As researchers and practitioners, we need to resist the practice of abandoning and neglecting this group of serially and variably traumatized men.

## Appendix

**Appendix:** Descriptive statistics for dependent and independent variables, adult male respondents.

Variables		N	Range	M(SD)
Dependent Variables				
	Depression treatment in prison	3,958	0–1	0.15(0.36)
	Depression symptoms	3,959	0–12	2.52 (2.95)
	Anxiety disorder treatment in prison	3,958	0–1	0.07(0.26)
	Anxiety symptoms	3,959	0–9	2.22 (2.48)
	Substance abuse treatment in prison	3,958	0–1	0.26 (0.44)
	Substance/alcohol in community	3,904	0–4	1.46(1.36)
	Interpersonal problems	3,904	0–7	1.81(2.01)
	Self-regulation problems	3,899	0–6	2.12(1.73)
	Anger-Buss-Perry Aggression Scale total score	3,949	12–60	28.5(11.2)
	Beck Hopelessness Scale	3,965	0–19	3.21(3.14)
Independent Variables				
Level-2, mean age		10	23.2–44.7	34.0(7.2)
Age		3,893	18–79	33.3(10.1)
White		3,898	0,1 (Y)	0.16 (0.37)
Latino		3,898	0,1 (Y)	0.25(0.43)
Other race		3,898	0,1 (Y)	0.08(0.27)
Education/GED		3,898	0,1 (Y)	0.61 (0.49)
Years served in prison since age 18		3,943	0–60	7.6 (6.7)
Current offense: Sex offense		3,956	0,1 (Y)	0.05 (0.22)
Current offense: Violent offense		3,956	0,1 (Y)	0.24 (0.43)
	Physical trauma < 18	3,895	0,1 (Y)	0.45 (0.50)
	Sexual trauma < 18	3,895	0,1 (Y)	0.11 (0.31)
	Physical trauma 18+	3,895	0,1 (Y)	0.31 (0.46)
	Sexual trauma 18+	3,895	0,1 (Y)	0.04 (0.21)
	Abandoned < 18	3,895	0,1 (Y)	0.20 (0.40)
	Abandoned 18+	3895	0,1 (Y)	0.18 (0.39)
